# Benign Solitary Fibrous Tumor of the Pleura

**DOI:** 10.7759/cureus.54111

**Published:** 2024-02-13

**Authors:** Seyedehtina Safaei, Ali Kimiaei, Pinar Çağan, Cemal Asim Kutlu

**Affiliations:** 1 Medicine, Bahcesehir University, Istanbul, TUR; 2 Thoracic Surgery, Bahcesehir University, Istanbul, TUR

**Keywords:** benign tumor, thoracic surgery, vats, pleural tumor, solitary fibrous tumor of the pleura

## Abstract

Solitary fibrous tumors of the pleura (SFTPs) are rare and typically benign neoplasms with limited reported cases. Despite their initial characterization as a pleura-based lesion, these neoplasms can occur in various anatomical locations. These tumors can present with paraneoplastic syndromes and have potential malignant transformations. Herein, we report a case of a 47-year-old female presenting with chest pain, cough, and weakness who was subsequently diagnosed with a benign SFTP. The patient required surgical intervention and underwent a wedge resection via video-assisted thoracoscopic surgery. The patient's recovery was uneventful, demonstrating effective management.

## Introduction

Solitary fibrous tumors of the pleura (SFTPs) are infrequent neoplasms derived from mesenchymal cells, with fewer than 1000 documented cases in the literature [1,2]. It is a slow-growing, localized fibrous tumor. Total en bloc resection is pivotal in treating a patient diagnosed with benign SFTP, which is linked to an excellent prognosis [3]. Despite being initially characterized as a pleura-based lesion, the SFTP has since been acknowledged as a widespread neoplasm that can manifest in various anatomical locations [4]. Moreover, it has been observed that benign SFTPs, the majority of which are pedunculated, exhibit a high cure rate and a low local recurrence rate, typically responding well to curative re-excision [5]. However, it is important to note that about 12% of SFTPs are malignant, and in rare cases, they have been identified as being associated with multiple paraneoplastic syndromes [6]. Therefore, it is crucial to consider the potential malignant features and paraneoplastic syndromes when diagnosing and managing SFTPs.

Nevertheless, the tumor commonly remains asymptomatic, progressively growing considerably. Consequently, this leads to the onset of obstructive and compressive symptoms such as dyspnea and coughing. Here, we present a 47-year-old woman diagnosed with benign SFTP.

## Case presentation

**Figure 1:**
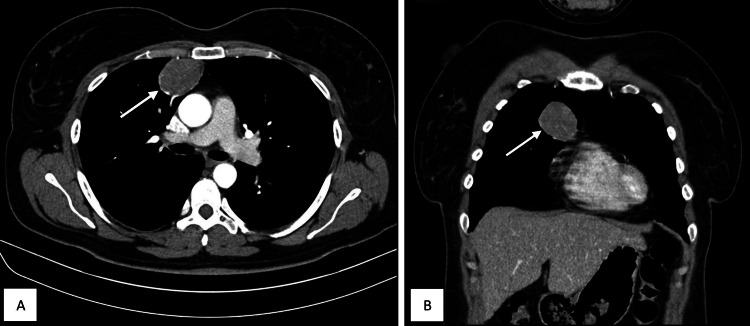
Axial (A) and coronal (B) CT scans showing a 5.5 x 5 x 2.5 cm mass (white arrow)

A 47-year-old woman presented with a month-long period of weakness and chest pain, which began following a severe bout of coughing. Prompted by these indications, a CT scan revealed a 5.5 x 5 x 2.5 cm mass (Figure 1).

Upon review of the diagnostic findings, the mass was ascertained to be noncancerous, prompting the suggestion of a right upper lobectomy. The patient underwent a wedge resection without any complications, conducted via video-assisted thoracoscopic surgery (VATS). A 3 cm incision was made at the 5th intercostal space along the midaxillary line on the right side, with the patient in the left lateral decubitus position.

**Figure 2:**
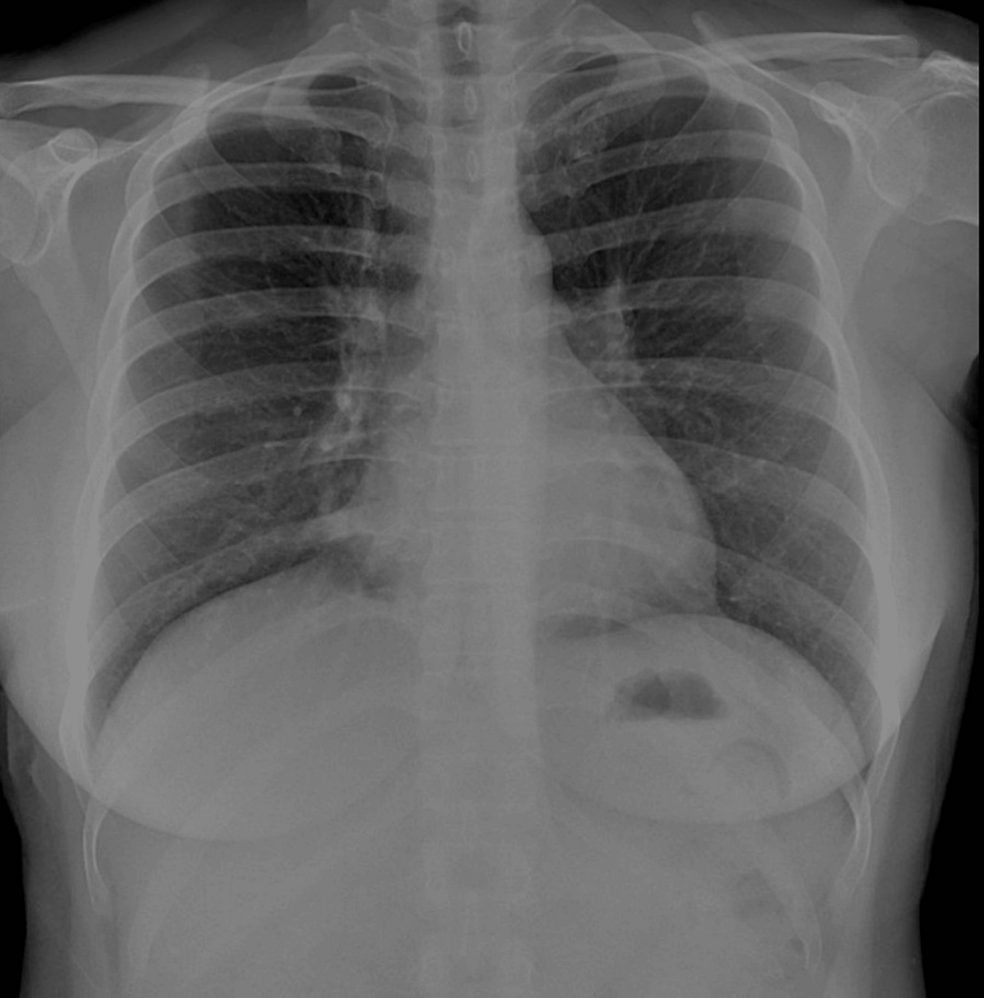
Postoperative chest X-ray confirming successful recovery

During the procedure, a solid, white-colored mass approximately 5 cm in size was observed in the anterior segment of the upper lobe through visual observation. A wedge resection was carried out using two staplers. A frozen section analysis indicated suspicion of noncancerous pathology with no signs of malignant cells. Thorough examinations for bleeding and leaks were performed, and a 20F polyethylene drain was inserted before closing the surgical site. The patient recuperated successfully and was discharged one day after the surgery. Subsequent postoperative chest x-rays and a follow-up appointment 10 days later confirmed a successful recovery (Figure 2).

Pathological examination of the biopsy material unveiled a tumor measuring 5.5 x 5 x 2.5 cm. Microscopic examination depicted a tumor characterized by intense collagen deposition with hyalinized vascular structures comprised of spindle and ovoid cells, exhibiting no mitotic activity or necrosis. Immunohistochemical findings revealed positivity for STAT6 and diffuse positivity for CD34, while PanCK was negative. Ki-67 staining indicated a low proliferative index of 1%, and smooth muscle actin (SMA) staining was negative. The final diagnosis was an SFTP located in the anterior region of the right upper lobe.

## Discussion

SFTPs represent a minority of primary pleural neoplasms, constituting less than 5% [7]. The etiology of SFTPs remains enigmatic, lacking established risk factors such as smoking, asbestos, or radiation [8]. These tumors are generally perceived as benign, well-circumscribed soft-tissue neoplasms; however, they manifest malignant characteristics in infrequent instances. Around 80% of SFTPs originate in the visceral pleura, while 20% arise in the parietal pleura [1]. Apart from their pleural origin, SFTPs have been documented in diverse atypical sites, including the peritoneum, mesentery, liver, pineal region, retroperitoneal space, kidney, and dura [9]. The biological behavior of SFTPs in these atypical locations remains poorly understood due to their rarity [4].

SFTPs predominantly affect individuals in their 6th and 7th decades, with no gender predilection [10]. They are often incidentally discovered on routine chest radiographs and can present asymptomatically, although some patients may experience nonspecific respiratory symptoms such as cough, dyspnea, chest pain, and hemoptysis [11]. Additionally, in the case of paraneoplastic syndromes, rare symptoms such as digital clubbing (Pierre-Marie-Bamberg) and recurrent hypoglycemic episodes (Doege-Potter) have been reported [12,13].

Computed tomography (CT) and magnetic resonance imaging (MRI) are frequently employed for visualizing the tumor and evaluating its characteristics. They typically present as pleural-based masses in CT imaging, displaying enhancement after contrast administration, often arising from the visceral pleura [1]. MRI can be useful in evaluating the extent of the tumor and its relationship to adjacent structures. Despite the usefulness of visualization, the definitive diagnosis is histopathological examination combined with a characteristic immunophenotype.

SFTPs exhibit a diverse cellular composition, featuring cells characterized by oval to spindle-shaped nuclei, minimal cytoplasm, and interspersed patternless collagen bands [14]. Immunohistochemically, SFTP typically shows positivity for vimentin, CD99, CD34, Bcl-2, and cytokeratin negativity [15].

The primary treatment approach for SFTP is surgical resection, with the completeness of the initial resection being crucial in preventing recurrence [3]. VATS has been used in the surgical treatment of pedunculated and free-moving tumors originating from the visceral pleura, demonstrating its effectiveness in managing such cases with a favorable long-term prognosis and a minimally invasive approach [16]. However, larger tumors may require thoracotomy. Interestingly, there are cases of larger masses excised successfully with VATS, proving its effectiveness in managing SFTPs [17,18]. The most favorable prognostic indicator is the complete surgical removal of the tumor, ensuring microscopically clear surgical margins [15].

In a study of 110 cases, the study revealed that the overall metastasis-free rates at 5 and 10 years were 74% and 55%, respectively [19]. Additionally, the disease-specific survival rates at 5 and 10 years were 89% and 73%, respectively. Notably, patient age, tumor size, and mitotic index emerged as predictors for both the time to metastasis and disease-specific mortality [19]. On the other hand, necrosis was a predictor of metastasis alone [19].

Benign tumors exhibit a low local recurrence rate, reported as low as 8% after complete resection [5]. In contrast, malignant SFTPs show a less enduring response, with up to 63% experiencing recurrence [5]. Long-term follow-up is warranted, with reports of late recurrence reported to occur as far out as 17 years following resection [14].

## Conclusions

In conclusion, our case adds to the scarce literature on benign SFTPs, offering valuable perspectives on effective diagnosis and treatment. The main treatment strategy for SFTPs is surgical resection via thoracotomy or the less invasive VATS. Further studies with a broader cohort of patients are needed to comprehensively evaluate the recurrence rates, postoperative complications, and overall survival rates between these surgical techniques.
